# Inhibition of Peptidyl Arginine Deiminase 4-Dependent Neutrophil Extracellular Trap Formation Reduces Angiotensin II-Induced Abdominal Aortic Aneurysm Rupture in Mice

**DOI:** 10.3389/fcvm.2021.676612

**Published:** 2021-07-30

**Authors:** Ming Wei, Xia Wang, Yanting Song, Di Zhu, Dan Qi, Shiyu Jiao, Guomin Xie, Ye Liu, Baoqi Yu, Jie Du, Yuji Wang, Aijuan Qu

**Affiliations:** ^1^Department of Physiology and Pathophysiology, School of Basic Medical Sciences, Capital Medical University, Beijing, China; ^2^Key Laboratory of Remodeling-Related Cardiovascular Diseases, Ministry of Education, Beijing, China; ^3^School of Pharmaceutical Sciences, Capital Medical University, Beijing, China; ^4^Beijing Institute of Heart, Lung and Blood Vessel Diseases, Beijing, China; ^5^Beijing Anzhen Hospital, Capital Medical University, Beijing, China

**Keywords:** abdominal aortic aneurysm, neutrophil extracellular traps, vascular smooth muscle cells, apoptosis, peptidyl arginine deiminase 4

## Abstract

**Objective:** Neutrophil infiltration plays an important role in the initiation and development of abdominal aortic aneurysm (AAA). Recent studies suggested that neutrophils could release neutrophil extracellular traps (NETs), leading to tissue injury in cardiovascular diseases. However, the role of NETs in AAA is elusive. This study aimed to investigate the role and underlying mechanism of NETs in AAA development.

**Methods and Results:** An angiotensin II (Ang II) infusion-induced AAA model was established to investigate the role of NETs during AAA development. Immunofluorescence staining showed that citrullinated histone 3 (citH3), myeloperoxidase (MPO), and neutrophil elastase (NE) (NET marker) expressions were significantly increased in Ang II-infused *ApoE*^−/−^ mice. The circulating double-stranded DNA (dsDNA) level was also elevated, indicating the increased NET formation during AAA. PAD4 inhibitor YW3-56 inhibited Ang II-induced NET formation. Disruption of NET formation by YW3-56 markedly reduced Ang II-induced AAA rupture, as revealed by decreased aortic diameter, vascular smooth muscle cell (VSMC) apoptosis, and elastin degradation. Apoptosis of VSMC was evaluated by TUNEL staining and Annexin V-FITC/PI staining through flow cytometry. Western blot and inhibition experiments revealed that NETs induced VSMC apoptosis *via* p38/JNK pathway, indicating that PAD4-dependent NET formation played an important role in AAA.

**Conclusions:** This study suggests that PAD4-dependent NET formation is critical for AAA rupture, which provides a novel potential therapeutic strategy for AAA disease.

## Introduction

Abdominal aortic aneurysm (AAA) is clinically defined as the increase of aortic diameter by at least 50% compared with the regular diameter. AAA is common in men aged over 65 years (1). In general, aneurysms remain asymptomatic until rupture. AAA rupture leads to a 30-day mortality rate up to 70% (2). Nowadays, surgical treatment is the most important strategy for treating AAA (3). However, the risk of surgery is high and long-term survival is still dissatisfactory (4, 5). Looking for more effective treatments for AAA requires a better understanding of its pathological process.

During AAA formation, major pathological changes include vascular smooth muscle cell (VSMC) apoptosis, extracellular matrix degradation, and inflammatory cell infiltration in aortic media and adventitia (6, 7). As the first line of innate immunity, neutrophils are quickly recruited to the location of inflammation (8). Neutrophils were associated with oxidative stress in mice and humans (9, 10). Neutrophil deficiency alleviated AAA (11). Therefore, neutrophils play an important role in AAA development.

In addition to phagocytosis and degranulation, neutrophils have also been found to form NETs in response to inflammatory stimuli. The extracellular structures of NETs are formed by DNA, decondensed chromatin, nuclear proteins, and cytoplasmic proteins (12, 13). NETosis is a program for the formation of NETs since it was initially shown that NET formation was accompanied by cell death (14). It is characterized by the release of decondensed chromatin associated with modification histone and granule components into the cytosol. Currently, different forms of NETosis have been described. Classical or suicidal NETosis leads to cell death. Vital NETosis releases NETs in the form of vesicles (15). In the formation of NETs, peptidyl arginine deiminase 4 (PAD4) mediates chromatin decondensation by citrullinating histones. Histone mixed with granule proteins is released from the neutrophil (16). As cytoplasmic proteins, NE and MPO can be transported into the nucleus to degrade linker histones, accelerating the process of chromatin decondensation (13, 17). Intensive investigations on NETs over the last years revealed a potential involvement of the NETs in immune diseases. NETs accelerated local pathological inflammatory response by increasing the infiltration of inflammatory factors and inducing the lytic cell death of VSMCs (18–20). The role of NETs in vascular diseases like thrombosis and atherosclerosis has been found. However, its role in AAA has been scarcely analyzed. It is important to clarify the underlying mechanism of NETs and find a more effective drug therapy to slow down the rupture process in AAA. Therefore, this study aims to investigate (1) the main mechanism of NETs in regulating the pathological process of AAA and (2) whether the AAA process can be inhibited by the administration of PAD4 inhibitor YW3-56.

In the current study, NET formation can be induced by Ang II. NETs induced the apoptosis of VSMCs *via* p38/JNK pathway. Furthermore, PAD4 inhibitor YW3-56 ameliorated AAA rupture by inhibiting NET formation. These results suggest that inhibition of the NET formation may provide a potential treatment method for AAA.

## Methods and Materials

### Mouse Model of Ang II-Induced AAA

*ApoE*^−/−^ mice on C57BL/6J background were purchased from the Jackson Laboratory (Bar Harbor, ME, USA). Mice were housed in temperature-controlled (20–22°C) and light-controlled (12-h light/dark cycle) rooms with free access to water and food. To establish the Ang II-induced AAA model, 10–12-week-old male mice were used for experiments. All mouse genotypes were identified by tail clip samples on PCR. Primers are listed in Supplementary Table 1. All animal studies were carried out in accordance with guidelines and approved by the Capital Medical University Animal Care and Use Committee. Experiments were performed under a project license (AEEI-2018-127) granted by the ethics board of Capital Medical University.

The Ang II-induced AAA model was generated as follows. Micro-osmotic pumps (ALZET DURECT 1004, Durect Corp, Cupertino, CA, USA) loaded with saline or angiotensin II (A9525, Sigma-Aldrich, St. Louis, MO, USA) were subcutaneously implanted in 10–12-week-old male *ApoE*^−/−^ mice at a delivery rate of 1,000 ng/kg/min for 28 days (21).

For NET inhibition *in vivo*, PAD4 inhibitor YW3-56 was synthesized as previously described (22). YW3-56 (10 mL/kg) or vehicle was administered intravenously every other day from 1 day before Ang II infusion. An equal amount of DMSO was used as the control group.

### Blood Pressure Measurements

Blood pressure was monitored every other day after Ang II infusion by the tail-cuff method (BP2000, Visitech Systems, Apex, NC, USA) to confirm the efficiency of Ang II as described previously (23).

### Vascular Ultrasonic Studies

Echocardiography assessment was performed to analyze the diameter of vessels in mice infused with Ang II after 28 days using a Vevo 2100 console (Visual Sonics Vevo 2100, FUJIFILM, Bothell, WA, USA). B-mode ultrasound data were acquired for the aorta in the diastole cardiac cycle. The transducer with a central frequency of 40 MHz and a focal length of 7 mm (MS 550D) was used. Images of the abdominal aorta of the mouse were acquired. The maximal dilated portion of the suprarenal aorta diameters was measured in internal diameter by ultrasonic statistical software analysis. The increase in the outer width of the suprarenal aorta by at least 50% was identified as aneurysm formation.

### Histological Analysis

All mice were sacrificed with inhaled isoflurane (1% in O_2_) at the end of the experiment, and the blood was collected before the mice were flushed with 20 mL saline-EDTA. Aortas were exposed under a dissecting microscope and photographed after the peri-adventitial tissue was removed. Subsequently, the abdominal aortas were obtained, fixed with 4% paraformaldehyde (PFA), and embedded in OCT. Aortas were sectioned into 7 μm for further analysis. Frozen aortic sections of *ApoE*^−/−^ mice were stained with hematoxylin (RY-ICH001a, Roby, Beijing, China) and eosin (RY-ICH002a, Roby, Beijing, China) (H&E) as described previously (23). Aortic sections were stained using the Gomori Aldehyde-Fuchsin Kit (MST-8047, Maixin Bio, Fuzhou, China) for elastin assessment as described previously. Elastin degradation was graded as (1) no degradation with a well-organized elastic layer; (2) mild degradation with some interruptions and fractures; (3) severe degradation with fragmentation and loss; and (4) rupture (24). At least five fields for each section were captured for elastic fiber content.

### Immunohistochemical Staining

After being washed by PBS three times, the frozen aortic sections were fixed with 4% PFA for 15 min and permeabilized with 0.1% Triton X-100 (T9284, Sigma, St.Louis, MO, USA) for 10 min. Samples were incubated with 3% hydrogen peroxide for 10 min followed by goat serum blocking (ZLI-9056, ZsBio, Beijing, China). The sections were incubated with the specific antibody for MPO (1:200, ab65871, Abcam, Cambridge, MA, USA) overnight at 4°C. After being washed with PBS three times the next day, the sections were incubated with goat anti-rabbit IgG secondary antibody (ZF-0136, ZsBio, Beijing, China) at room temperature for 30 min. The positive-stained cells were detected with diaminobenzidine (DAB) (GK600505, GeneTech, Shanghai, China).

### Immunofluorescence Staining

For immunofluorescence analysis, cells grown on fibronectin-coated glass coverslips or frozen aortic sections were fixed with 4% PFA for 15 min, permeabilized with 0.1% Triton X-100, and blocked with 10% goat serum at room temperature for 1 h. Samples were incubated with anti-rabbit citH3 antibody (for citrulline R2, R8, and R17; 1:200, ab5103, Abcam, USA), anti-rat Ly6G (1:200, ab25377, Abcam, USA), anti-rabbit-NE antibody (1:200, ab21595, Abcam, USA), and anti-rabbit-MPO antibody (1:200, ab65871, Abcam, USA) at 4°C overnight. On the 2nd day, slides were incubated with goat anti-rabbit/rat TRITC/FITC secondary antibody for 1 h at room temperature and mounted with DAPI (4′,6-diamidino-2-phenylindole, Vector, ZsBio, Beijing, China). Images were obtained using a confocal microscope. At least five fields for each coverslip were captured. The image analysis was performed with ImageJ (NIH, Bethesda, MD, USA).

### TUNEL Staining

After rinsing twice with PBS, the samples were fixed with 4% PFA and permeabilized in 0.1% Triton X-100 for 10 min. After being washed with PBS, the samples were blocked by goat serum for 30 min at room temperature. A TUNEL reaction mixture (about 10 μL) (12156792910, Roche, Balsai, Switzerland) was added to the samples. After being incubated at 37°C for 1.5 h, samples were incubated with anti-SM22α antibody (1:200, ab7817, Abcam, USA) at 4°C overnight. Samples were incubated with goat anti-rabbit FITC secondary antibody for 1 h at room temperature the next day and sealed with DAPI.

### Neutrophil Isolation

Bone marrow-derived neutrophils were isolated from *ApoE*^−/−^ mice. Bone marrow cells were collected by flushing the femur with PBS and filtering through a sterile 70-μm nylon cell strainer. Cell suspension was layered in a ratio of 1 to 3 on top of Histopaque-1077 (10771, Sigma-Aldrich, MO, USA). After centrifugation, cell precipitation was resuspended with PBS. Cell suspension was layered in a ratio of 1 to 2 on top of Histopaque-1119 (11191, Sigma-Aldrich, MO, USA). Neutrophils were separated on the top of Histopaque-1119 after centrifugation. Neutrophils were resuspended in RPMI 1640 medium after being washed with PBS (18). Neutrophil purity was checked by immunofluorescence. In addition, 1 × 10^5^ cells/well were seeded in 2% fibronectin-treated coverslips to perform the immunofluorescence staining. Cells were incubated for 4 h at 37°C.

### Preparation for NETs

Mouse bone marrow neutrophils were stimulated with Ang II (5 μM) for 4 h to induce NETs. After confirmation of NET formation by visualization of extracellular DNA with DAPI, the culture medium was removed and neutrophils were washed and incubated for 1 h with RPMI 1640 medium. Supernatants were collected and centrifuged (10 min, 400 g). Neutrophils and cell debris were pelleted at the bottom. Supernatant was spun (10 min, 17,000 g) at 4°C. Pellets were resuspended and obtained together in ice-cold PBS. Quant-iT PicoGreen dsDNA Assay Kit (Thermo Fisher Scientific, Waltham, MA, USA) was used to measure the concentration according to the manufacturer's instructions, and NETs were either used immediately or stored at −80°C (25).

### Mouse Primary VSMC Isolation

Primary VSMCs were isolated from aortas of 10- to 12-week-old male *ApoE*^−/−^ mice as previously described (23). Mice were killed by inhaled isoflurane (1% in O_2_). The whole aortic portions were dissected and then digested with 347 U/mg type II collagenase (Worthington, Lakewood, NJ, USA) at 37°C for 30 min. The adventitia was carefully removed. A sterile cotton-tipped applicator was used to remove the endothelium by gently rubbing the intima. The aortas were minced into small pieces and digested with a mixture of 347 U/mg collagenase II and 6 U/mg elastase IV (Sigma-Aldrich) for 30 min at 37°C. Cells were then cultured with SMCM (ScienCell, CA, USA) supplemented with 10% fetal bovine serum (FBS), 1% smooth muscle growth supplement, and 1% penicillin streptomycin. VSMCs from passages 3 to 9 were used for all *in vitro* experiments. VSMCs were treated with 10 μg NETs for 24 h before starvation (1% FBS) for 8 h (26). To determine whether p38 inhibitor SB203580 or JNK inhibitor SP600125 could reverse NET-induced VSMC apoptosis, p38 inhibitor SB203580 (S1076, Selleckchem, Houston, TX, USA) or JNK inhibitor SP600125 (S1876, Beyotime, Shanghai, China) was preincubated in primary VSMC.

### Annexin V/PI Assay

Annexin V/PI apoptosis detection kit (556547, BD Biosciences, San Jose, CA, USA) was used to detect apoptosis according to the manufacturer's instructions. The cell culture medium of the primary VSMCs was carefully collected into a centrifuge tube. Then, the VSMCs were digested with EDTA-free trypsin and collected in the centrifuge tube again, and centrifuged at 1,000 rpm for 5 min. Cells were washed with PBS and resuspended with 1× binding buffer. Annexin V/Alexa Fluor 488 of 5 μl was added into the cell suspension and mixed well. After being kept at room temperature for 5 min, PI (5 μL) was added and mixed with cell suspension. The mixtures were analyzed immediately using flow cytometry.

### Real-Time Quantitative PCR

Total RNA was extracted from the whole aortic portion of mice using TRIzol reagent (15596018, Invitrogen, Carlsbad, CA, USA). Retro-transcription in cDNA was synthesized from 2 μg of RNA and performed with GoScript™ Reverse Transcription System (A5001, Promega, Mannheim, Germany). Real-time quantitative PCR was performed with SYBR Green PCR Kit (420A, Takara, Shiga, Japan) with the CFX Connect Real-Time System (Bio-Rad, Hercules, CA, USA). Results were analyzed, and fold change over *Actb* was calculated by the comparative cycle method (2^−ΔΔCt^). All primer sequences are shown in Supplementary Table 2.

### Western Blot

Whole-cell lysate was extracted from VSMCs or neutrophils using RIPA Lysis Buffer (C1053, Applygen, Beijing, China). The protein concentration of the samples was measured by Pierce™ BCA Protein Assay Kit (23209, Thermo Fisher Scientific, Waltham, MA, USA). An equal amount of protein per sample and molecular weight markers was subjected to SDS-polyacrylamide gel electrophoresis (SDS-PAGE). Membranes were blocked and probed for primary antibodies against citH3 (1:1000 dilution; ab5103, Abcam, USA), GAPDH (1:2000 dilution; 10494-1-AP, Proteintech, Chicago, IL, USA), p38 (1:1000 dilution; 8690s, CST, Danvers, MA, USA), p-p38 (Thr180/Tyr182) (1:1000 dilution; 4511s, CST, USA), JNK (1:1000 dilution; 9252s, CST, USA), and p-JNK (Thr183/Tyr185) (1:1000 dilution; 4668s, CST, USA) overnight at 4°C. Anti-mouse (1:2000, 7076S; Cell Signaling, MA, USA) and anti-rabbit secondary antibody (1:2000, 7074S; Cell Signaling, MA, USA) conjugated with horseradish peroxidase for 1 h at room temperature. Chemiluminescence HRP Substrate Kit (Millipore, Bedford, MA, USA) was used to perform immunodetection analysis. The mean band intensity was measured with ImageJ.

### Statistical Analysis

All data are shown as the means ± S.E.M. Two-tailed unpaired Student's *t*-test was used to compare two groups. Comparisons among more than two independent groups were analyzed by one-way or two-way analysis of variance (ANOVA). Differences among multiple groups with one variable were determined using one-way analysis of variance (one-way ANOVA) followed by Bonferroni's *post-hoc* test. Two-way ANOVA followed by Bonferroni's *post-hoc* test was used to compare multiple groups with more than one variable. The Fisher exact test was used to compare the incidence and rupture rate of AAA. The log-rank (Mantel–Cox) test was used to analyze the survival percentage of Ang II-infused mice. Statistical analysis was performed with Prism 7 software (GraphPad Software, San Diego, CA, USA). *p* < 0.05 was considered statistically significant.

## Results

### Ang II Induced NET Formation *in vivo*

**Figure 1:**
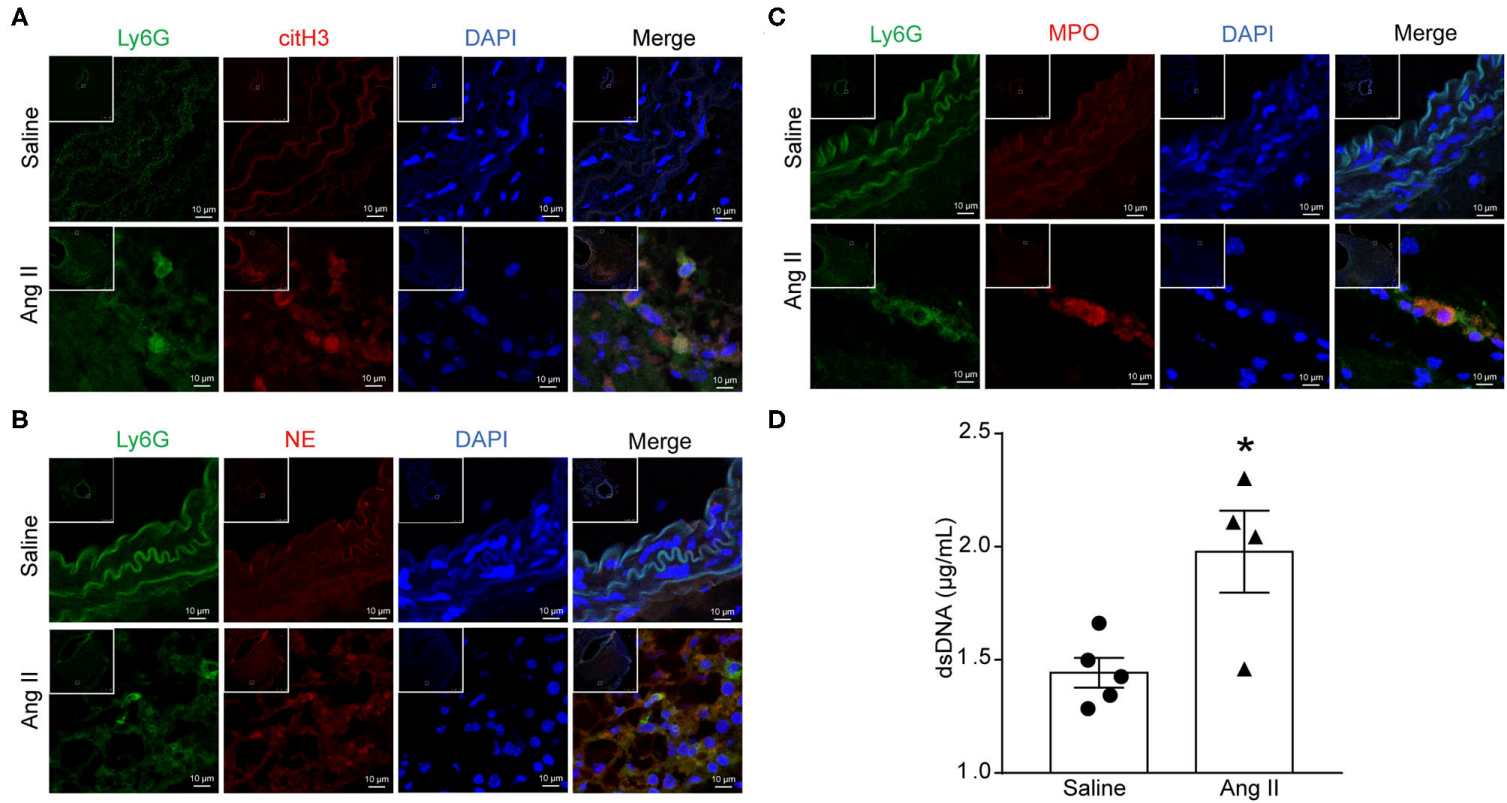
Ang II induced NET formation *in vivo*. *ApoE*^−/−^ mice were infused with saline or 1,000 ng/kg/min Ang II for 28 days. **(A–C)** Immunofluorescence analysis of mouse abdominal aortas for NET markers citH3 (red), NE (red), and MPO (red) with neutrophil marker Ly6G (green). Nuclei were stained with DAPI. **(D)** Measurement of dsDNA in the serum from saline or Ang II (1,000 ng/kg/min) infused *ApoE*^−/−^ mice. *n* = 5/saline group, *n* = 4/Ang II group, *p < 0.05. Statistical significance was analyzed by the unpaired *t*-test.

To determine the formation of NETs in Ang II-induced AAA, *ApoE*^−/−^ mice were infused with saline or 1,000 ng/kg/min Ang II for 28 days to establish the Ang II-induced AAA. PAD4-mediated decondensation of chromatin to form citrullinated histones is a key step in the formation of NETs. Therefore, the presence of citH3 is a marker of NETs (27). Cytoplasmic proteins NE and MPO were also used as NET markers. Immunofluorescence results showed that citH3 (red), neutrophil elastase (red), or MPO (red) colocalized with DAPI in Ly6G (green)-marked neutrophils in the Ang II-treated AAA (Figures 1A–C). dsDNA release associated with the formation of NETs (28) and was used as one of the detection indicators for NETs (26). The dsDNA level was also increased in Ang II-infused mice compared with the saline-treated group (Figure 1D). These results showed that NET formation is increased in Ang II-induced AAA in *ApoE*^−/−^ mice.

### Ang II Induced NET Formation *in vitro*

**Figure 2:**
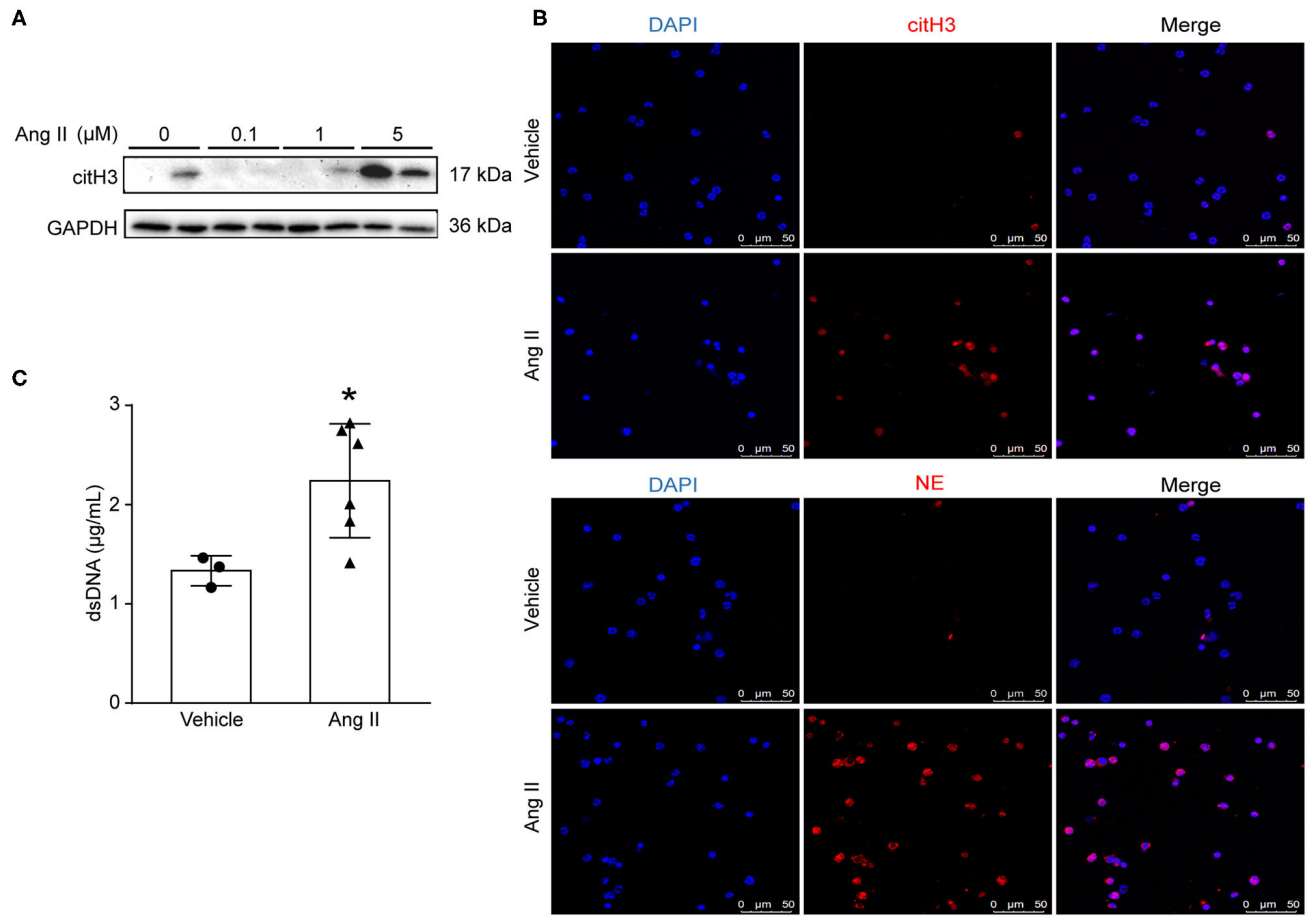
Ang II induced NET formation *in vitro*. Neutrophils were isolated from *ApoE*^−/−^ mice and treated with Ang II for 4 h. **(A)** Western blot for citH3 in different concentrations of Ang II (0, 100 nM, 1 μM, and 5 μM)-treated neutrophils. **(B)** Immunofluorescence analysis of Ang II (5 μM)-treated bone marrow-derived neutrophils for citH3 (red) or NE (red). Nuclei were stained with DAPI. **(C)** Measurement of dsDNA in Ang II-treated neutrophils. *n* = 3/vehicle group, *n* = 6/Ang II group, *p < 0.05. Statistical significance was analyzed by the unpaired *t*-test.

To examine whether Ang II could induce NET formation *in vitro*, bone marrow-derived neutrophils were isolated by density gradient centrifugation from *ApoE*^−/−^ mice and stimulated with Ang II with dosages of 0, 100 nM, 1 μM, and 5 μM *in vitro*. The expression of citH3 was detected by Western blot (Figure 2A). The results showed that citH3 expression was significantly increased after 5 μM Ang II stimulation in neutrophils. Immunofluorescence staining results also showed that Ang II significantly increased the expression of citH3 and NE compared with the vehicle group (Figure 2B). The dsDNA level was also increased in Ang II stimulated bone-marrow neutrophils (Figure 2C). These results demonstrated that Ang II induced NET formation *in vitro*.

### PAD4 Inhibitor YW3-56 Suppressed Ang II-Induced NET Formation *in vitro*

**Figure 3:**
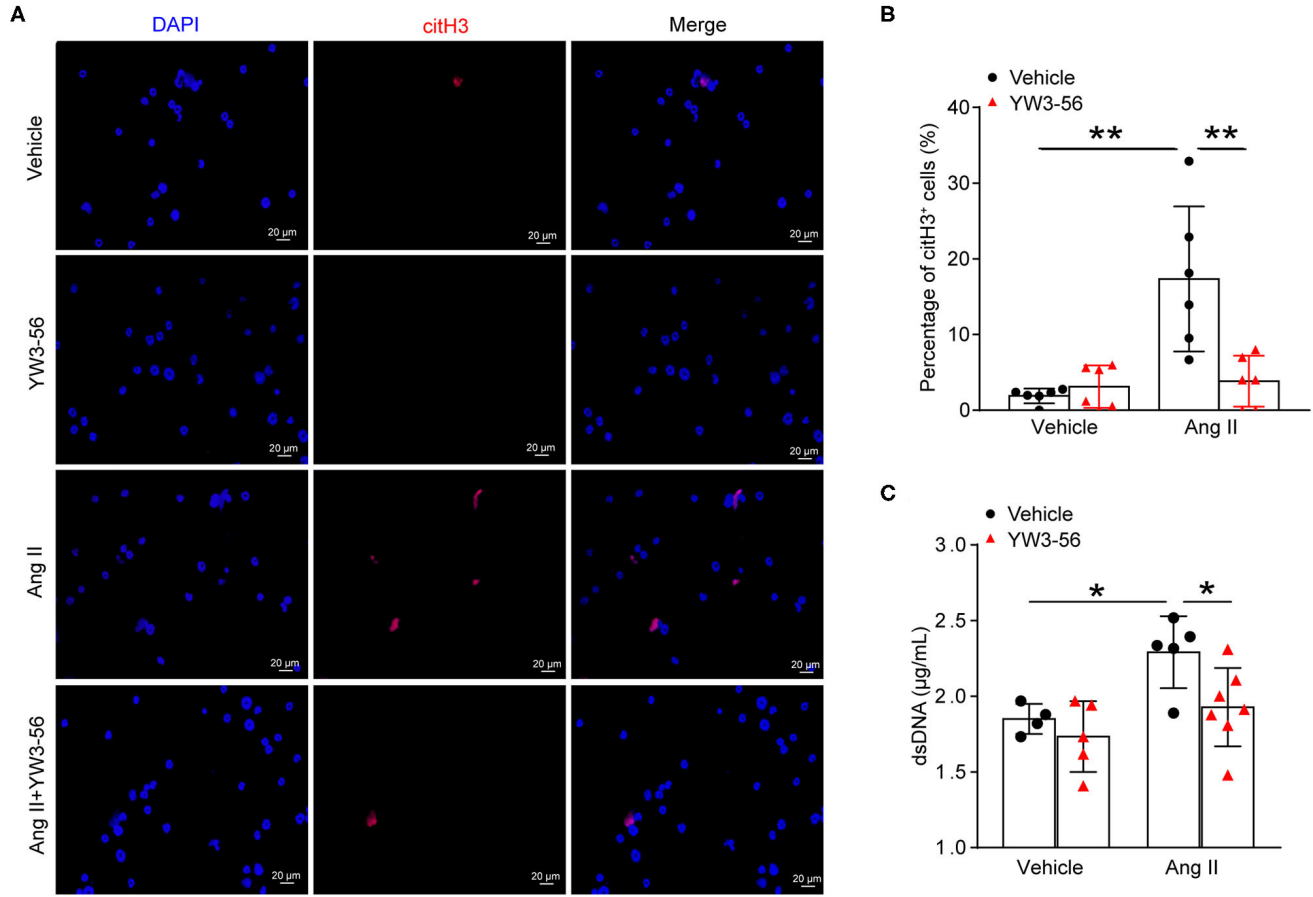
PAD4 inhibitor YW3-56 suppressed Ang II-induced NET formation *in vitro*. Bone marrow-derived neutrophils from *ApoE*^−/−^ mice were treated with vehicle or 5 μM Ang II for 4 h in the presence of vehicle or YW3-56. **(A)** Immunofluorescence analysis for citH3 (red). Nuclei were stained with DAPI. **(B)** Measurement of the percentage of citH3-positive cells. *n* = 6 per group, **p < 0.01. **(C)** Measurement of dsDNA in vehicle or Ang II-stimulated neutrophils with or without YW3-56 treatment. *n* = 4/vehicle group, *n* = 5/YW3-56 group, *n* = 5/Ang II group, *n* = 7/Ang II+YW3-56 group, *p < 0.05. Statistical significance was determined by one-way ANOVA test followed by the unpaired *t*-test.

NET formation was increased in AAA. PAD4-mediated histone citrullination and chromatin decondensation are essential steps in NET formation. Cl-amidine is a known PAD pan-inhibitor. To develop more potent inhibitors of PAD4, the N-terminus and C-terminus of Cl-amidine were modified. The activity of the synthetic compound YW3-56 was evaluated. YW3-56 as a Cl-amidine analog has improved the bioavailability and increased the permeability. Moreover, subsequent trials showed that YW3-56 exerted an antitumor effect (22). To investigate whether YW3-56 could inhibit the formation of NETs, immunofluorescence staining was performed in bone marrow-derived neutrophils with Ang II stimulation. The results showed that Ang II significantly increased the expression of citH3, whereas this effect was decreased by YW3-56 treatment (Figures 3A,B). Nevertheless, YW3-56 slightly reduced the dsDNA level stimulated by Ang II in bone marrow-derived neutrophils (Figure 3C). The above results showed that YW3-56 reduced the formation of NETs in mouse bone marrow-derived neutrophils induced by Ang II.

### PAD4 Inhibitor YW3-56 Reduced Ang II-Induced AAA Rupture

**Figure 4:**
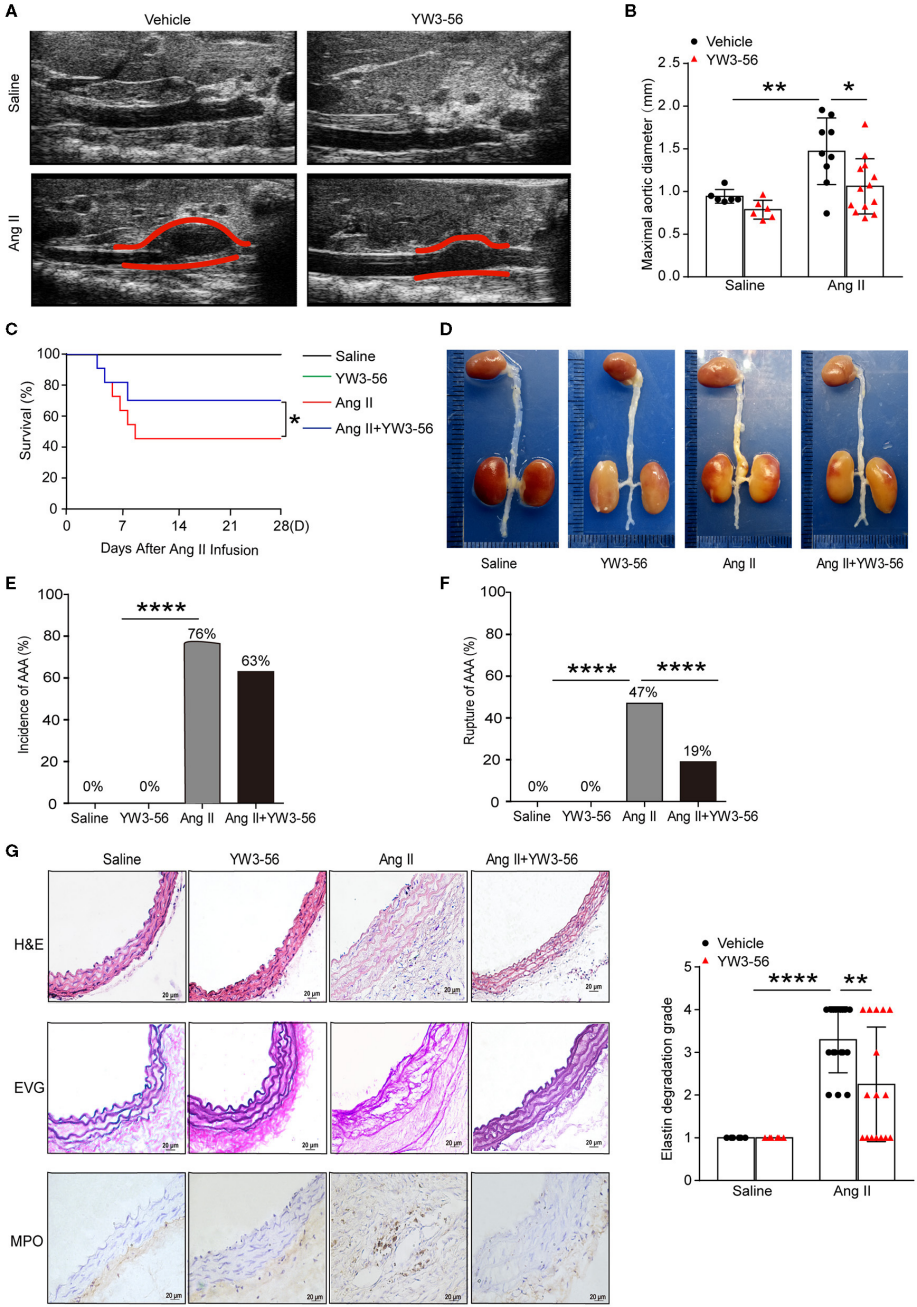
PAD4 inhibitor YW3-56 reduced Ang II-induced AAA rupture. *ApoE*^−/−^ mice were infused with saline or 1,000 ng/kg/min Ang II for 28 days in the presence of vehicle or YW3-56. **(A)** B-mode ultrasound of abdominal aortas and **(B)** statistical analysis of maximal aortic diameter. *n* = 6/saline group, *n* = 6/YW3-56 group, *n* = 9/Ang II group, *n* = 13/Ang II+YW3-56 group, *p < 0.05, **p < 0.01. Statistical significance was determined by one-way ANOVA test followed by the unpaired *t*-test. **(C)** The survival rate of Ang II-induced AAA in *ApoE*^−/−^ mice in the presence of vehicle or YW3-56. *p < 0.05, significance was determined by the log-rank (Mantel–Cox) test. **(D)** Representative photos for aortas from the above mice. **(E)** The incidence rate of AAA from the above mice. *n* = 6–17, ****p < 0.0001. Statistical significance was determined by Fisher exact test. **(F)** The rupture rate of AAA from the above mice. *n* = 6–17, ****p < 0.0001. Statistical significance was determined by Fisher exact test. **(G)** Representative images of H&E, EVG, and MPO staining for the abdominal aortas from the above mice. *n* = 6–17, **p < 0.01, ****p < 0.0001. Statistical significance was determined by one-way ANOVA test followed by the unpaired *t*-test.

To validate the effect of YW3-56 in Ang II-induced AAA of *ApoE*^−/−^ mice, YW3-56 (10 mL/kg) was intraperitoneally injected every other day from 1 day before Ang II infusion. Ultrasound results showed that Ang II significantly increased the blood vessel diameter leading to the formation of AAA, while YW3-56 restricted the blood vessel diameter dilation and reduced the formation of AAA induced by Ang II (Figures 4A,B). YW3-56 also reduced the mortality induced by Ang II in *ApoE*^−/−^ mice (Figure 4C). Morphological results showed that YW3-56 attenuated the Ang II-induced increase of the vessel diameter (Figure 4D). The incidence rate of AAA in Ang II infused mice with or without YW3-56 showed no significant difference (Figure 4E). The rupture rate with YW3-56 treatment in Ang II infused mice was significantly decreased compared with Ang II infused mice (Figure 4F). These data suggested that PAD4 inhibitor YW3-56 inhibited AAA rupture but has little effect on AAA initiation/formation. Morphologically, H&E staining of the abdominal aorta of *ApoE*^−/−^ mice showed that YW3-56 mitigated the blood vessel diameter and inflammatory cell infiltration increase caused by Ang II. Gomori Aldehyde-Fuchsin results showed that Ang II significantly increased the fracture of the elastic lamina, which can be significantly alleviated by YW3-56. MPO immunohistochemistry showed that YW3-56 reduced neutrophil infiltration caused by Ang II perfusion (Figure 4G). These results showed that PAD4 inhibitor YW3-56 mainly reduced the blood vessel diameter, inflammation, and elastin degradation induced by Ang II. It emphasized the important role of YW3-56 in suppressing AAA rupture.

### PAD4 Inhibitor YW3-56 Decreased NET Formation and VSMC Apoptosis in Ang II-Induced AAA

**Figure 5:**
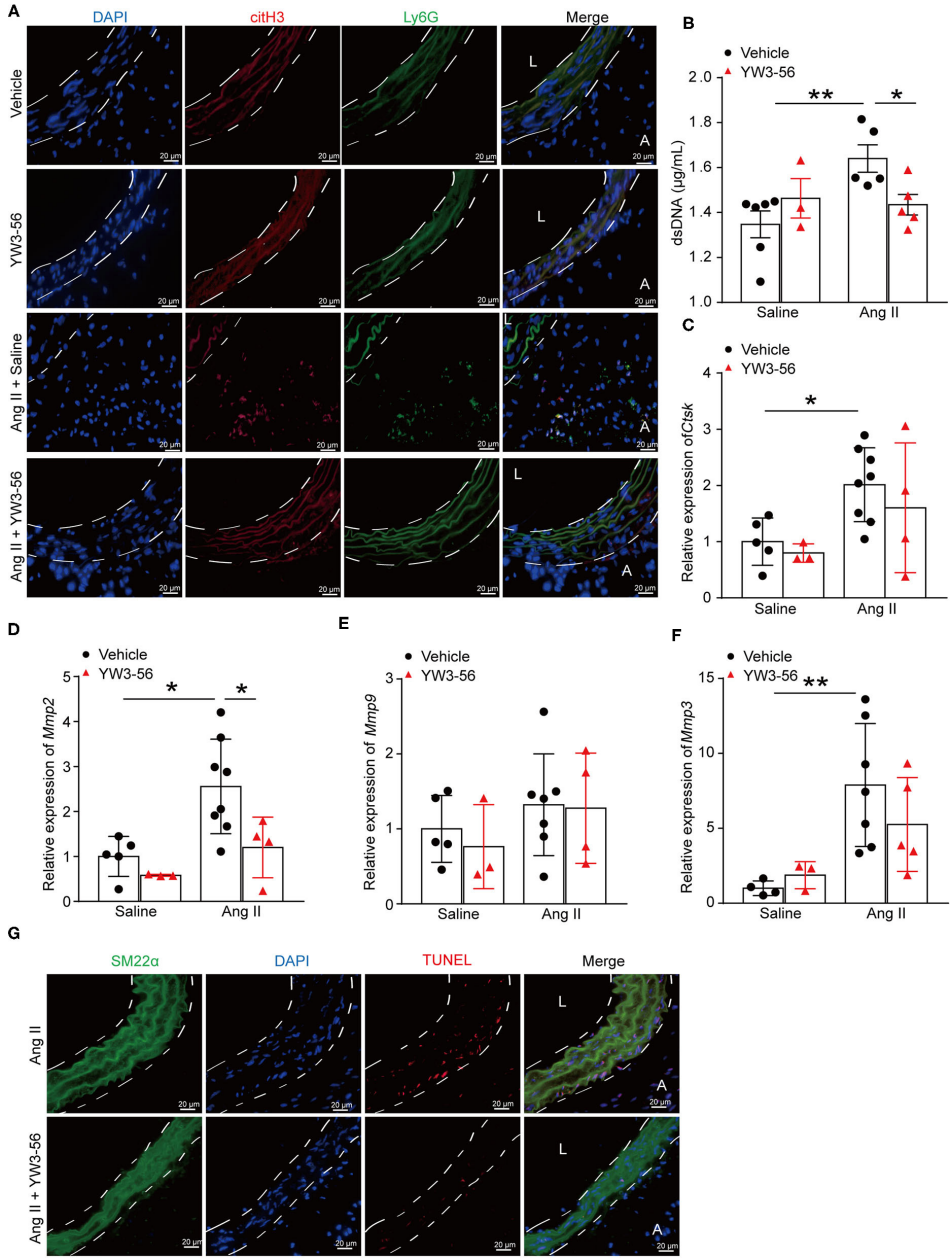
PAD4 inhibitor YW3-56 reduced NET formation and VSMC apoptosis in Ang II-induced AAA. *ApoE*^−/−^ mice were infused with saline or 1,000 ng/kg/min Ang II for 28 days in the presence of vehicle or YW3-56. **(A)** Immunofluorescence analysis for citH3 (red) and Ly6G (green) in the abdominal aortas. Nuclei were stained with DAPI. L: lumen, A: adventitia. **(B)** Measurement of dsDNA in the serum of Ang II-induced *ApoE*^−/−^ mice with or without YW3-56. *n* = 6/saline group, *n* = 3/YW3-56 group, *n* = 5/Ang II group, *n* = 5/Ang II+YW3-56 group, *p < 0.05, **p < 0.01. **(C–F)** Aortic *Mmp2/3/9* and *Ctsk* mRNAs were measured by RT-qPCR. *n* = 3–8, *p < 0.05, **p < 0.01. **(G)** TUNEL staining for apoptosis cells (red). VSMCs were stained with SM22α. Nuclei were stained with DAPI. Statistical significance was determined by one-way ANOVA test followed by the unpaired *t*-test.

To further clarify whether YW3-56 reduced the rupture of AAA by inhibiting the formation of NETs, immunofluorescence staining was performed. The results showed that YW3-56 reduced the citH3 level in Ang II-induced aneurysm (Figure 5A). YW3-56 also decreased the dsDNA level induced by Ang II (Figure 5B). RT-qRCR results showed that Ang II infusion increased the level of *Ctsk, Mmp2, Mmp9*, and *Mmp3*, while YW3-56 attenuated the *Mmp2* expression induced by Ang II (Figures 5C–F). Immunofluorescence staining also showed that YW3-56 decreased the apoptosis of VSMCs induced by Ang II (Figure 5G). These results showed that YW3-56 reduced VSMC apoptosis and further decreased AAA rupture by suppression of NET formation.

### NET Induced VSMC Apoptosis *via* p38/JNK Pathway

**Figure 6:**
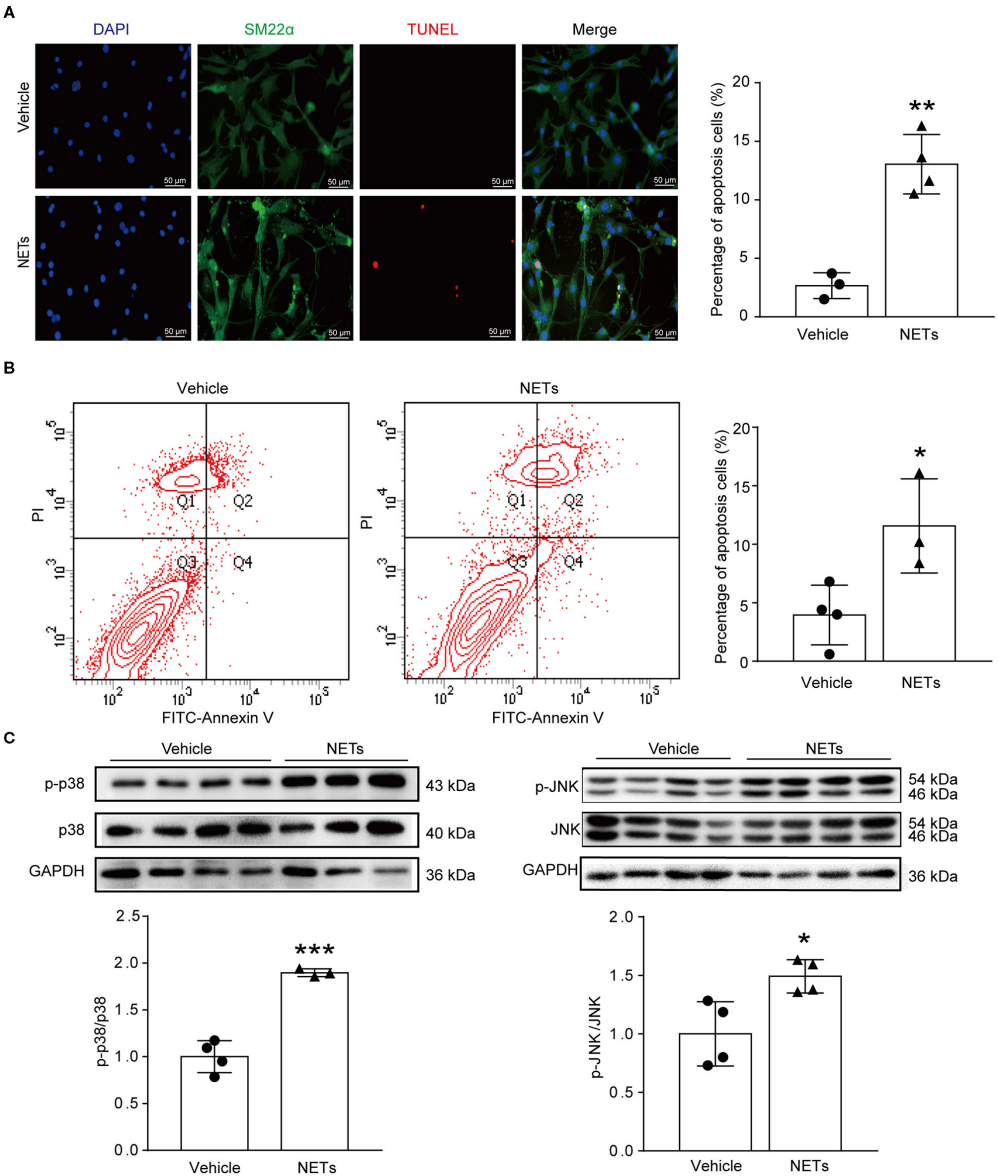
NETs induced VSMC apoptosis *via* p38/JNK pathway. Primary VSMCs isolated from aortas of *ApoE*^−/−^ mice were treated with vehicle or 10 μg NETs for 24 h. **(A)** TUNEL staining for apoptosis cells (red). The percentage of apoptosis cells was analyzed. VSMCs were stained with SM22α. Nuclei were stained with DAPI. *n* = 3/vehicle group, *n* = 4/NET group, **p < 0.01. Statistical significance was analyzed by the unpaired *t*-test. **(B)** Annexin V-FITC/PI staining for VSMC apoptosis. *n* = 4/vehicle group, *n* = 3/NET group, *p < 0.05. Statistical significance was analyzed by the unpaired *t*-test. **(C)** Western blot for p38/JNK level. *n* = 3–4, *p < 0.05, ***p < 0.001. Statistical significance was analyzed by the unpaired *t*-test.

VSMC apoptosis leads to massive loss of contractile cells and dilation of the abdominal aortic wall (29). To explore whether NETs induced by Ang II affected the apoptosis of VSMCs, NETs were used to stimulate VSMCs for 24 h. TUNEL staining showed that NETs significantly increased the apoptosis of VSMCs (Figure 6A). Furthermore, Annexin V-FITC/PI staining flow cytometry analysis results also demonstrated that NET treatment increased VSMC apoptosis (Figure 6B). Mitogen-activated protein kinases are widely expressed serine/tyrosine kinases that play an important role in a variety of signal transduction pathways in mammalian cells (30). The p38 signaling pathway is an important branch of the MAPK pathway which is essential in various physiological and pathological processes such as apoptosis, inflammation, cell stress, and cell cycle and growth (30). To study whether NETs induced VSMC apoptosis *via* the MAPK signaling pathway, Western blot was performed on VSMCs after NET stimulation. The results showed that the phosphorylation levels of p38 and JNK were significantly higher after NET administration (Figure 6C), suggesting that NETs induced VSMC apoptosis *via* p38/JNK pathway.

### Inhibition p38/JNK Pathway Suppressed NET-Induced VSMC Apoptosis

**Figure 7:**
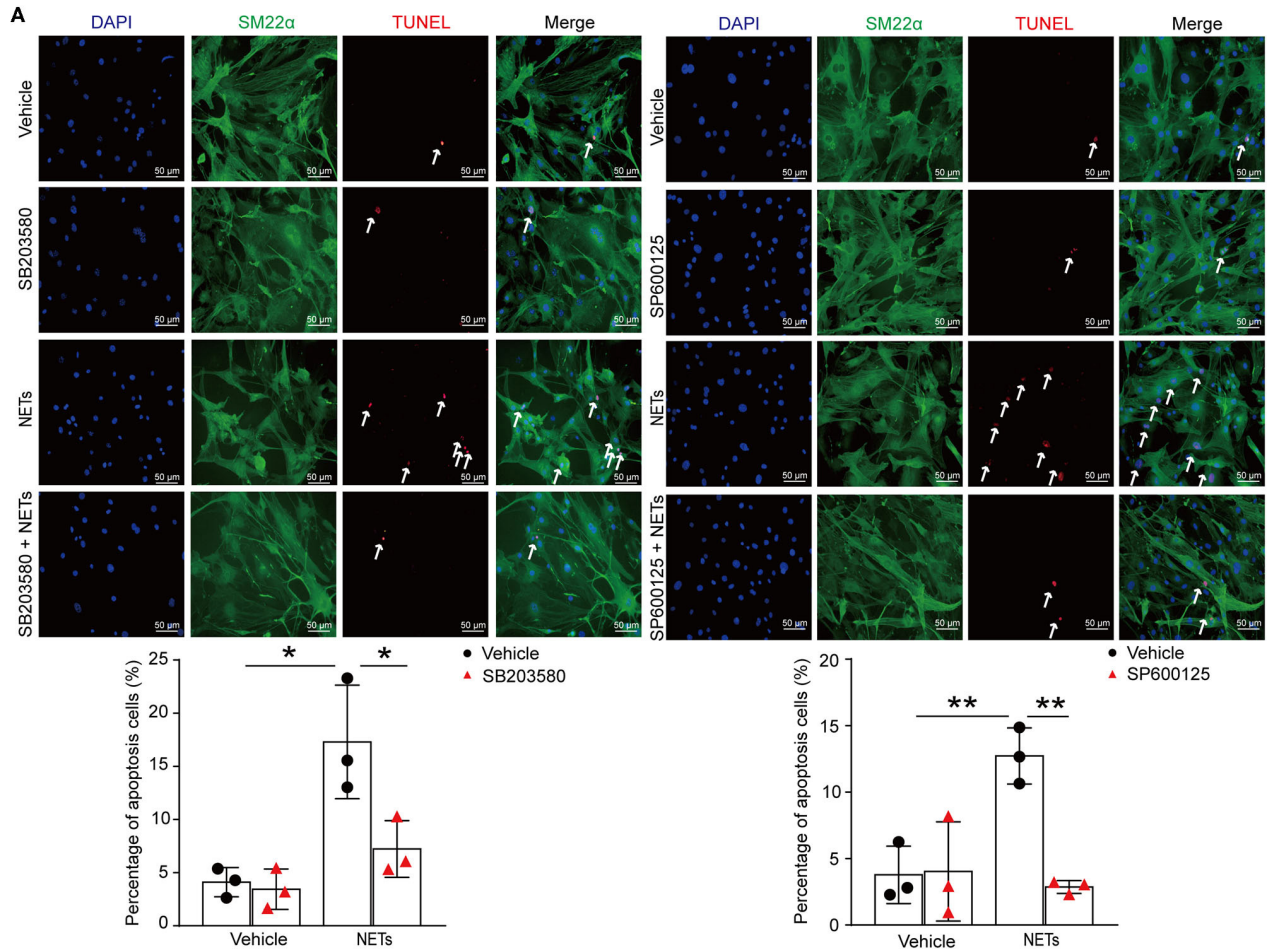
Inhibition p38/JNK pathway suppressed NETs induced VSMC apoptosis. Primary VSMCs isolated from aortas of *ApoE*^−/−^ mice were preincubated with p38 inhibitor SB203580 or JNK inhibitor SP600125 for 30 min before NET treatment for 24 h. **(A)** TUNEL staining for apoptosis cells (red) and the percentage of apoptosis cells were analyzed. VSMCs were stained with SM22α. Nuclei were stained with DAPI. *n* = 3 per group, *p < 0.05, **p < 0.01. Statistical significance was determined by one-way ANOVA test followed by the unpaired *t*-test.

To verify whether NETs induced VSMC apoptosis *via* p38/JNK pathway, VSMCs were preincubated with p38 inhibitor SB203580 or JNK inhibitor SP600125 for 30 min before NET treatment. TUNEL staining showed that the inhibitor of p38/JNK could decrease NET-induced VSMC apoptosis (Figure 7A). These results suggested that NETs accelerated the apoptosis of VSMCs through p38 and JNK signaling pathway.

## Discussion

In summary, this work provided several new findings as follows: (1). NETs accelerated VSMC apoptosis *via* p38/JNK pathway; (2). PAD4 inhibitor YW3-56 inhibited NET formation and then reduced Ang II-induced aortic diameter increase and elastin degradation. It is important to note that PAD4 inhibitor YW3-56 significantly alleviates Ang II-induced AAA rupture. Inhibition of NET formation or p38/JNK pathway may therefore be a potential therapeutic strategy for treating AAA.

AAA rupture is thought to occur when the hemodynamics generated by pulsating blood flow exceeds the strength of the AAA wall. The process involves several pathological processes, including infiltration of inflammatory cells, ECM degradation, and VSMC apoptosis. Neutrophils contribute to the pathological mechanisms of AAA, such as proteolysis, oxidative stress, and adventitia immune inflammatory processes driving aortic wall remodeling (31). NETs are considered not only an important defense tool of neutrophils but also an indispensable participant to exacerbate a variety of cardiovascular diseases, such as atherosclerosis and atherothrombosis (32). NETs were associated with AAA development in the elastase-induced AAA model. DNAse I premature treatment suppressed AAA development, suggesting the involvement of NETs in AAA formation (19). Pretreatment with ceramide synthesis fumonisin inhibitor decreased IL-1β-induced ceramide synthesis and IL-1β-induced NETosis, indicating that ceramide synthesis is involved in NETosis in AAA (18). In this study, Ang II treatment significantly increased the formation of NETs. This is consistent with a previous report that levels of citH3 increased in the Ang II-induced AAA model (33). However, the mechanism by which NET affect AAA processes is not clear.

It is well-established that VSMCs play an important role in the development of AAA (34). miR-106a promoted VSMC apoptosis and MMP production. Therefore, the development of AAA was accelerated (35). In this study, TUNEL staining and Annexin V-FITC/PI analysis results also demonstrated that NET treatment increased VSMC apoptosis *via* p38/JNK pathway. ECM degradation causes elastin degradation, thereby impairing aortic strength and elasticity (36). MMPs contain several members implicated in AAA formation. MMPs are capable of degrading elastin type I and type III collagen which are predominant types found in the aortic wall. MMP overexpression was also responsible for aneurysmal degeneration and rupture in experimental studies in rats (37, 38). NET formation increased the level of MMP2/9 in this study. Targeting ECM degradation, VSMC apoptosis, and inflammation may be a potential therapeutic approach for AAA pathogenesis.

PAD4 plays an important role in NET formation by catalyzing histone citrullination (16). PAD inhibitor Cl-amidine reduced NET formation in atherosclerosis induced by high-cholesterol diet (39). Therefore, reducing NET formation by inhibiting PAD4 is a way to treat AAA. PAD4 inhibitor YW3-56 reduced Ang II-induced aortic diameter increase and elastin degradation. PAD4 inhibitor YW3-56 significantly alleviates Ang II-induced AAA rupture.

Of note, Wolf Eilenberg et al. (40) demonstrated that histone citrullination is a novel biomarker in AAA and PAD4 inhibitor GSK484 attenuated Ang II-induced AAA. However, how the NETs affected AAA was unexplored. In our study, NETs increased apoptosis of VSMCs *via* p38/JNK pathway. In addition, PAD4 inhibitor YW3-56 as a Cl-amidine analog has improved the bioavailability and increased the permeability. YW3-56 suppressed cancer cell growth significantly (22). Inhibition of NET formation by YW3-56 reduced the rupture of AAA. It provided in-depth knowledge of a new PAD4 inhibitor YW3-56 in decreasing NET formation.

In conclusion, this study showed that NET formation was increased in Ang II-induced AAA. Inhibition of NET formation by YW3-56 ameliorated Ang II-induced AAA rupture. Clarifying the role of NETs in AAA may contribute to a better understanding of the pathophysiology of AAA, which also provides a new idea for the prevention and treatment in AAA.

## Data Availability Statement

The raw data supporting the conclusions of this article will be made available by the authors, without undue reservation.

## Ethics Statement

The animal study was reviewed and approved by AEEI-2018-127.

## Author Contributions

MW performed the experiments and wrote the manuscript. XW guided the drawing. YS, DZ, and DQ discussed the results. YW generously provided PAD4 inhibitor YW3-56. AQ designed the experiments and wrote the manuscript. All the authors read and modified the final version of the manuscript.

## Conflict of Interest

The authors declare that the research was conducted in the absence of any commercial or financial relationships that could be construed as a potential conflict of interest.

## Publisher's Note

All claims expressed in this article are solely those of the authors and do not necessarily represent those of their affiliated organizations, or those of the publisher, the editors and the reviewers. Any product that may be evaluated in this article, or claim that may be made by its manufacturer, is not guaranteed or endorsed by the publisher.

## Abbreviations

AAA: Abdominal aortic aneurysm
Ang II: Angiotensin II
*ApoE*: Apolipoprotein E
citH3: Citrullinated histones3
MPO: Myeloperoxidase
NETs: Neutrophil extracellular traps
NE: Neutrophil elastase
PAD4: Peptidyl arginine deiminase 4
PBS: Phosphate-buffered saline
VSMCs: Vascular smooth muscle cells
IL: Interleukin
TF: Tissue factor
MMP: Matrix metalloproteinases
TUNEL: TdT-mediated dUTP nick end labeling
qPCR: Quantitative-polymerase chain reaction
PBS: Phosphate-buffered saline
H&E: Hematoxylin and eosin staining
FBS: Fetal bovine serum
DAPI: 4′,6-diamidino-2-phenylindole
SM22α: Smooth Muscle 22 alpha
GAPDH: Glyceraldehyde-3phosphate dehydrogenase
JNK: Jun N-terminal kinase.

## References

[1] Keisler B Carter C. Abdominal aortic aneurysm. Am Fam Physician. (2015) 91:538–43. 25884861

[2] Tchana-Sato V Sakalihasan N Defraigne JO. Ruptured abdominal aortic aneurysm. Rev Med Liege. (2018) 73:296–9. 10.3238/arztebl.2012.0727 29926569

[3] Lindeman JH Matsumura JS. Pharmacologic management of aneurysms. Circ Res. (2019) 124:631–46. 10.1161/CIRCRESAHA.118.312439 30763216 PMC6386187

[4] O'Driscoll JM Bahia SS Gravina A Di Fino S Thompson MM Karthikesalingam A. Transthoracic echocardiography provides important long-term prognostic information in selected patients undergoing endovascular abdominal aortic repair. Circ Cardiovasc Imaging. (2016) 9:e003557. 10.1161/CIRCIMAGING.115.003557 26860969

[5] Buck DB van Herwaarden JA Schermerhorn ML Moll FL. Endovascular treatment of abdominal aortic aneurysms. Nat Rev Cardiol. (2014) 11:112–23. 10.1038/nrcardio.2013.196 24343568 PMC4128641

[6] Nordon M Hinchliffe RJ Loftus IM Thompson MM. Pathophysiology and epidemiology of abdominal aortic aneurysms. Nat Rev Cardiol. (2011) 8:92–102. 10.1038/nrcardio.2010.180 21079638

[7] Raaz U Toh R Maegdefessel L Adam M Nakagami F Emrich FC. Hemodynamic regulation of reactive oxygen species: implications for vascular diseases. Antioxid Redox Signal. (2014) 20:914–28. 10.1089/ars.2013.5507 23879326 PMC3924901

[8] Weber C Zernecke A Libby P. The multifaceted contributions of leukocyte subsets to atherosclerosis: lessons from mouse models. Nat Rev Immunol. (2008) 8:802–15. 10.1038/nri2415 18825131

[9] Pagano MB Zhou HF Ennis TL Wu X Lambris JD Atkinson JP. Complement-dependent neutrophil recruitment is critical for the development of elastase-induced abdominal aortic aneurysm. Circulation. (2009) 119:1805–13. 10.1161/CIRCULATIONAHA.108.832972 19307471 PMC2758616

[10] Houard X Touat Z Ollivier V Louedec L Philippe M Sebbag U. Mediators of neutrophil recruitment in human abdominal aortic aneurysms. Cardiovasc Res. (2009) 82:532–41. 10.1093/cvr/cvp048 19201759 PMC2682614

[11] Eliason JL Hannawa KK Ailawadi G Sinha I Ford JW Deogracias MP. Neutrophil depletion inhibits experimental abdominal aortic aneurysm formation. Circulation. (2005) 112:232–40. 10.1161/CIRCULATIONAHA.104.517391 16009808

[12] Brinkmann V Reichard U Goosmann C Fauler B Uhlemann Y Weiss DS. Neutrophil extracellular traps kill bacteria. Science. (2004) 303:1532–5. 10.1126/science.1092385 15001782

[13] Vorobjeva NV Pinegin BV. Neutrophil extracellular traps: mechanisms of formation and role in health and disease. Biochemistry (Mosc). (2014) 79:1286–96. 10.1134/S0006297914120025 25716722

[14] Steinberg BE Grinstein S. Unconventional roles of the NADPH oxidase: signaling, ion homeostasis, cell death. Sci STKE. (2007) 2007:pe11. 10.1126/stke.3792007pe11 17392241

[15] de Bont CM Boelens WC Pruijn GJM. NETosis, complement, and coagulation: a triangular relationship. Cell Mol Immunol. (2019) 16:19–27. 10.1038/s41423-018-0024-0 29572545 PMC6318284

[16] Neeli I Dwivedi N Khan S Radic M. Regulation of extracellular chromatin release from neutrophils. J Innate Immun. (2009) 1:194–201. 10.1159/000206974 20375577 PMC6951038

[17] Karlsson A Dahlgren C. Assembly and activation of the neutrophil NADPH oxidase in granule membranes. Antioxid Redox Signal. (2002) 4:49–60. 10.1089/152308602753625852 11970843

[18] Meher K Spinosa M Davis JP Pope N Laubach VE Su G. Novel role of IL (Interleukin)-1beta in neutrophil extracellular trap formation and abdominal aortic aneurysms. Arterioscler Thromb Vasc Biol. (2018) 38:843–53. 10.1161/ATVBAHA.117.309897 29472233 PMC5864548

[19] Yan H Zhou HF Akk A Hu Y Springer LE Ennis TL. Neutrophil proteases promote experimental abdominal aortic aneurysm *via* extracellular trap release and plasmacytoid dendritic cell activation. Arterioscler Thromb Vasc Biol. (2016) 36:1660–9. 10.1161/ATVBAHA.116.307786 27283739 PMC4965335

[20] Silvestre-Roig C Braster Q Wichapong K Lee EY Teulon JM Berrebeh N. Externalized histone H4 orchestrates chronic inflammation by inducing lytic cell death. Nature. (2019) 569:236–40. 10.1038/s41586-019-1167-6 31043745 PMC6716525

[21] Qin Y Cao X Guo J Zhang Y Pan L Zhang H. Deficiency of cathepsin S attenuates angiotensin II-induced abdominal aortic aneurysm formation in apolipoprotein E-deficient mice. Cardiovasc Res. (2012) 96:401–10. 10.1093/cvr/cvs263 22871592 PMC3500043

[22] Wang Y Li P Wang S Hu J Chen XA Wu J. Anticancer peptidylarginine deiminase (PAD) inhibitors regulate the autophagy flux and the mammalian target of rapamycin complex 1 activity. J Biol Chem. (2012) 287:25941–53. 10.1074/jbc.M112.375725 22605338 PMC3406678

[23] Qi Wei M Jiao S Song Y Wang X Xie G. Hypoxia inducible factor 1alpha in vascular smooth muscle cells promotes angiotensin II-induced vascular remodeling *via* activation of CCL7-mediated macrophage recruitment. Cell Death Dis. (2019) 10:544. 10.1038/s41419-019-1757-0 31320613 PMC6639417

[24] Chen HZ Wang F Gao P Pei JF Liu Y Xu TT. Age-associated sirtuin 1 reduction in vascular smooth muscle links vascular senescence and inflammation to abdominal aortic aneurysm. Circ Res. (2016) 119:1076–88. 10.1161/CIRCRESAHA.116.308895 27650558 PMC6546422

[25] Najmeh S Cools-Lartigue J Giannias B Spicer J Ferri LE. Simplified human neutrophil extracellular traps (NETs) isolation and handling. J Vis Exp. (2015) 98:52687. 10.3791/52687 25938591 PMC4541576

[26] Li L Yu X Liu J Wang Z Li C Shi J. Neutrophil extracellular traps promote aberrant macrophages activation in behcet's disease. Front Immunol. (2020) 11:590622. 10.3389/fimmu.2020.590622 33633724 PMC7901995

[27] Du M Yang W Schmull S Gu J Xue S. Inhibition of peptidyl arginine deiminase-4 protects against myocardial infarction induced cardiac dysfunction. Int Immunopharmacol. (2020) 78:106055. 10.1016/j.intimp.2019.106055 31816575

[28] Toussaint M Jackson DJ Swieboda D Guedan A Tsourouktsoglou TD Ching YM. Host DNA released by NETosis promotes rhinovirus-induced type-2 allergic asthma exacerbation. Nat Med. (2017) 23:681–91. 10.1038/nm.4332 28459437 PMC5821220

[29] Zhang Z Zou G Chen X Lu W Liu J Zhai S. Knockdown of lncRNA PVT1 inhibits vascular smooth muscle cell apoptosis and extracellular matrix disruption in a murine abdominal aortic aneurysm model. Mol Cells. (2019) 42:218–27. 10.14348/molcells.2018.0162 30726659 PMC6449717

[30] Sun Y Liu WZ Liu T Feng X Yang N Zhou HF. Signaling pathway of MAPK/ERK in cell proliferation, differentiation, migration, senescence and apoptosis. J Recept Signal Transduct Res. (2015) 35:600–4. 10.3109/10799893.2015.1030412 26096166

[31] Kim HW Blomkalns AL Ogbi M Thomas M Gavrila D Neltner BS. Role of myeloperoxidase in abdominal aortic aneurysm formation: mitigation by taurine. Am J Physiol Heart Circ Physiol. (2017) 313:H1168–79. 10.1152/ajpheart.00296.2017 28971841 PMC5814655

[32] Doring Y Soehnlein O Weber C. Neutrophil extracellular traps in atherosclerosis and atherothrombosis. Circ Res. (2017) 120:736–43. 10.1161/CIRCRESAHA.116.309692 28209798

[33] Spinosa M Su G Salmon MD Lu G Cullen JM Fashandi AZ. Resolvin D1 decreases abdominal aortic aneurysm formation by inhibiting NETosis in a mouse model. J Vasc Surg. (2018) 68:93S–103S. 10.1016/j.jvs.2018.05.253 30470363 PMC6459013

[34] Kawai T Takayanagi T Forrester SJ Preston KJ Obama T Tsuji T. Vascular ADAM17 (a Disintegrin and Metalloproteinase Domain 17) is required for angiotensin II/beta-aminopropionitrile-induced abdominal aortic aneurysm. Hypertension. (2017) 70:959–63. 10.1161/HYPERTENSIONAHA.117.09822 28947615 PMC5679456

[35] Han ZL Wang HQ Zhang TS He YX Zhou H. Up-regulation of exosomal miR-106a may play a significant role in abdominal aortic aneurysm by inducing vascular smooth muscle cell apoptosis and targeting TIMP-2, an inhibitor of metallopeptidases that suppresses extracellular matrix degradation. Eur Rev Med Pharmacol Sci. (2020) 24:8087–95. 10.26355/eurrev_202008_22493 32767336

[36] Krishna SM Morton SK Li J Golledge J. Risk factors and mouse models of abdominal aortic aneurysm rupture. Int J Mol Sci. (2020) 21:7250. 10.3390/ijms21197250 33008131 PMC7583758

[37] Rabkin SW. The role matrix metalloproteinases in the production of aortic aneurysm. Prog Mol Biol Transl Sci. (2017) 147:239–65. 10.1016/bs.pmbts.2017.02.002 28413030

[38] Maguire M Pearce SWA Xiao R Oo AY Xiao Q. Matrix metalloproteinase in abdominal aortic aneurysm and aortic dissection. Pharmaceuticals (Basel). (2019) 12:118. 10.3390/ph12030118 31390798 PMC6789891

[39] Knight JS Luo W O'Dell AA Yalavarthi S Zhao W Subramanian V. Peptidylarginine deiminase inhibition reduces vascular damage and modulates innate immune responses in murine models of atherosclerosis. Circ Res. (2017) 114:947–56. 10.1161/CIRCRESAHA.114.303312 24425713 PMC4185401

[40] Eilenberg W Zagrapan B Bleichert S Ibrahim N Knobl V Brandau A. Histone citrullination as a novel biomarker and target to inhibit progression of abdominal aortic aneurysms. Transl Res. (2021) 233:32–46. 10.1016/j.trsl.2021.02.003 33571683

