# Supplementary material for: Inhibition of Peptidyl Arginine Deiminase 4-Dependent Neutrophil Extracellular Trap Formation Reduces Angiotensin II-Induced Abdominal Aortic Aneurysm Rupture in Mice
Source: Front Cardiovasc Med. 2021 Jul 30;8:676612. doi: 10.3389/fcvm.2021.676612 (PMC8360833; doi:10.3389/fcvm.2021.676612)
Supplement: Supplementary file 1 [file Table_1.DOCX]

Supplemental Table 1

The list of primers used in genotype is as follows:

| Primer | Sequences (5’-3’) |
| --- | --- |
| oIMR0180 | GCC TAG CCG AGG GAG AGC CG |
| oIMR0181 | TGT GAC TTG GGA GCT CTG CAG C |
| oIMR0182 | GCC GCC CCG ACT GCA TCT |

Supplemental Table 2

The list of primers used in RT-qRCR is as follows:

| Primer | Sequences (5’-3’) |
| --- | --- |
| *Mmp2* | F: TCAACGGTCGGGAATACAGC |
|  | R: CCATGGTAAACAAGGCTTCATGG |
| *Mmp9* | F: TGGTCTTCCCCAAAGACCTG |
|  | R: AGCGGTACAAGTATGCCTCTG |
| *Mmp3* | F: CCCTGGGACTCTACCACTCA |
|  | R: GCTGTGGGAGTTCCATAGAGG |
| *Ctsk* | F: CACCCTTAGTCTTCCGCTCA |
|  | R: CTTGAACACCCACATCCTGCT |
| *Actb* | F: TTCTTTGCAGCTCCTTCGTT |
|  | R: ATGGAGGGGAATACAGCCC |
